# High-throughput screening for natural compound-based autophagy modulators reveals novel chemotherapeutic mode of action for arzanol

**DOI:** 10.1038/s41419-021-03830-5

**Published:** 2021-05-31

**Authors:** Jana Deitersen, Lena Berning, Fabian Stuhldreier, Sara Ceccacci, David Schlütermann, Annabelle Friedrich, Wenxian Wu, Yadong Sun, Philip Böhler, Niklas Berleth, María José Mendiburo, Sabine Seggewiß, Ruchika Anand, Andreas S. Reichert, Maria Chiara Monti, Peter Proksch, Björn Stork

**Affiliations:** 1 Institute of Molecular Medicine I, Medical Faculty and University Hospital Düsseldorf, Heinrich-Heine-University Düsseldorf, Universitätsstraße 1, 40225 Düsseldorf, Germany; 2 Department of Pharmacy, University of Salerno, Via Giovanni Paolo II 132, 84084 Salerno, Fisciano Italy; 3 Institute of Biochemistry and Molecular Biology I, Medical Faculty and University Hospital Düsseldorf, Heinrich-Heine-University Düsseldorf, Universitätsstraße 1, 40225 Düsseldorf, Germany; 4 Institute of Pharmaceutical Biology and Biotechnology, Faculty of Mathematics and Natural Sciences, Heinrich-Heine-University Düsseldorf, Universitätsstraße 1, 40225 Düsseldorf, Germany

**Keywords:** Macroautophagy, Target identification

## Abstract

Autophagy is an intracellular recycling pathway with implications for intracellular homeostasis and cell survival. Its pharmacological modulation can aid chemotherapy by sensitizing cancer cells toward approved drugs and overcoming chemoresistance. Recent translational data on autophagy modulators show promising results in reducing tumor growth and metastasis, but also reveal a need for more specific compounds and novel lead structures. Here, we searched for such autophagy-modulating compounds in a flow cytometry-based high-throughput screening of an in-house natural compound library. We successfully identified novel inducers and inhibitors of the autophagic pathway. Among these, we identified arzanol as an autophagy-modulating drug that causes the accumulation of ATG16L1-positive structures, while it also induces the accumulation of lipidated LC3. Surprisingly, we observed a reduction of the size of autophagosomes compared to the bafilomycin control and a pronounced accumulation of p62/SQSTM1 in response to arzanol treatment in HeLa cells. We, therefore, speculate that arzanol acts both as an inducer of early autophagosome biogenesis and as an inhibitor of later autophagy events. We further show that arzanol is able to sensitize RT-112 bladder cancer cells towards cisplatin (CDDP). Its anticancer activity was confirmed in monotherapy against both CDDP-sensitive and -resistant bladder cancer cells. We classified arzanol as a novel mitotoxin that induces the fragmentation of mitochondria, and we identified a series of targets for arzanol that involve proteins of the class of mitochondria-associated quinone-binding oxidoreductases. Collectively, our results suggest arzanol as a valuable tool for autophagy research and as a lead compound for drug development in cancer therapy.

## Introduction

The discovery of approved anticancer drugs falls behind the rapid increase in cancer-related mortality. Cancer drug discovery, therefore, focusses on repurposing approved and identifying novel bioactive compounds that can serve as lead compounds. Most drugs developed over the last decades are based on natural compounds^1,2^. Their advantages lie in the inexhaustible quantity of compounds available in nature, their often non-synthesizable complexity, and a high degree of stereochemistry^3^. They often target evolutionarily conserved pathways that regulate cell fate decisions such as apoptosis, necrosis, senescence, or autophagy.

Autophagy is a lysosomal pathway with a mainly cytoprotective purpose that plays a role in different human pathologies such as neurodegeneration or cancer^4–7^. The main characteristic of (macro-)autophagy is the sequestration of aggregates, long-lived proteins, or damaged organelles by an autophagosomal membrane and its subsequent fusion with the lysosome for the degradation of bulk or selected cargo. Autophagy is basally active in most cell types, but can also be induced by growth factor or nutrient deficiency, hypoxia, aggregates, or mitochondrial damage^8–10^. Dependent on the stimulus, specific autophagy receptors help to eliminate sources of damage such as bacterial or viral components in xenophagy or damaged mitochondria in mitophagy.

In canonical autophagy, 5’-AMP-activated protein kinase (AMPK)-dependent activation and mammalian target of rapamycin (mTOR)-dependent inhibition regulate the autophagy-initiating unc-51-like kinase 1 (ULK1) complex. Upon induction of autophagy, the ULK1 complex activates the class III phosphatidylinositol 3-kinase (PIK3C3/VPS34) complex that produces phosphatidylinositol 3-phosphate (PI3P) at subregions of the ER^11^. ATG9A as well as the PI3P-binding proteins DCFP1 and WIPI1/2 are involved in membrane elongation and recruit the ubiquitin-like ATG12—ATG5-ATG16L1 complex. Together with ATG7 and ATG3, the ATG12—ATG5-ATG16L1 complex conjugates phosphatidyl-ethanolamine to LC3 (ref. ^12^). Lipidated LC3 decorates autophagosomal membranes and serves as an “anchor” for the recruitment of autophagosomal cargo or downstream autophagy regulators. For instance, LC3 binds p62 (also known as sequestosome 1, SQSTM1), which is an autophagic receptor for ubiquitinated cargo^13^. After fusion of autophagosome and lysosome, lysosomal hydrolases degrade not only autophagic cargo but also LC3 and LC3-bound receptors such as p62/SQSTM1. Accordingly, a reduction in both LC3 and p62/SQSTM1 levels serves as a readout for autophagic flux.

Autophagy ensures cell survival and prevents the accumulation of carcinogenic stimuli in cells, suggesting a protective role of autophagy against tumorigenesis. In late-stage cancer, on the other hand, autophagy can be tumor-promoting due to its catabolic function, supporting solid tumors in hypoxic regions and enabling the tumor microenvironment to contribute nutrients and growth factors^14–18^. In these tumors, autophagy has been found to contribute to cancer cell survival and poor outcome^19,20^. Recent attempts in translational cancer research, therefore, investigate the inhibition of autophagy in both monotherapy and combinational treatment in order to sensitize cancer cells to chemotherapy^21–24^. In contrast, inducers of autophagy are also discussed as potential chemotherapeutics, driving cancer cells into autophagy-associated or autophagic cell death^25–29^.

This work investigates an in-house library of natural compounds in order to find modulators of autophagy, which can be applied as novel chemotherapeutics for monotherapy or combinational therapy. We found that arzanol, a phloroglucinol derivative isolated from the plant *Helichrysum italicum* (Asteraceae), impairs the viability of bladder cancer cells, and we investigated its molecular mode of action. We identify arzanol as dual modulator of autophagy and expand the list of arzanol targets by mitochondria-related oxidoreductases and autophagy-related proteins. Taken together, we propose arzanol as a new tool to study autophagy and as a potential lead structure for combinational chemotherapy with CDDP in urothelial bladder carcinoma cells.

## Results

### High-throughput screening reveals modulators of autophagy

**Fig. 1:**
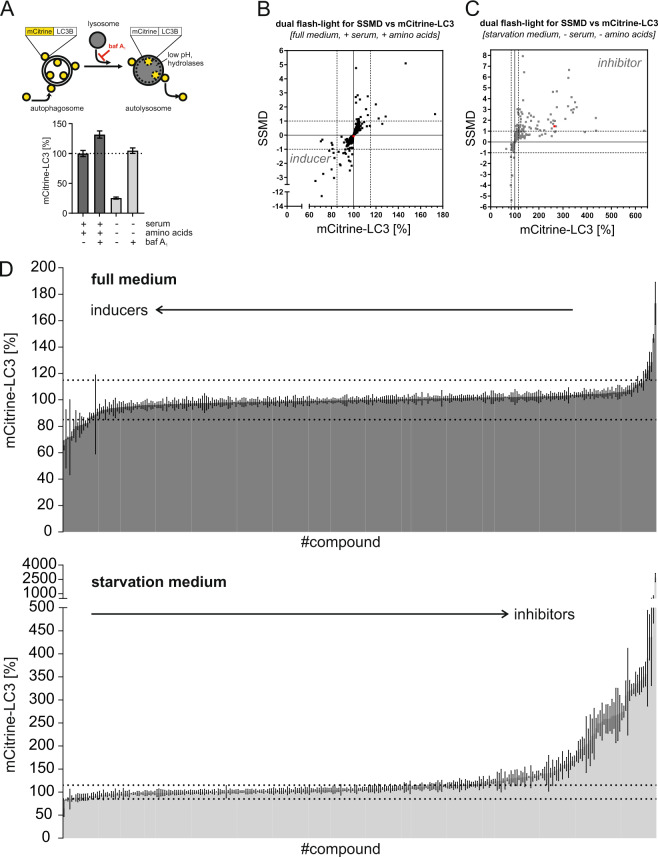
High-throughput screening for natural compound-based autophagy modulators. **A** Detection of starvation-induced and bafilomycin A_1_-inhibited autophagy in a flow cytometric screening. MEFs stably expressing mCitrine-LC3 were cultured either in full medium plus serum or in starvation medium and were treated with either 10 nM bafilomycin A_1_ or mock treatment (DMSO) for 6 h (*n* ≥ 19). The mCitrine fluorescence is shown as a percentage relative to that of mock-treated cells. Data represent means ± SEM. **B**, **C** High-throughput autophagy screening of 300 natural compounds. The diagram shows dual-flashlight plots for strictly standardized mean difference (SSMD) versus the average percentage of mCitrine-LC3. Dotted lines define potential inducers and inhibitors of autophagy. Cells were treated with 10 µM of each compound for 6 h (*n* = 3). Arzanol is highlighted in red. **D** The diagram shows individual levels of mCitrine-LC3 fluorescence from (**B**, **C**) for each of the 300 compounds in full or starvation medium. Dotted lines mark the ±15% difference in mCitrine-LC3 levels. Data represent means ± SEM.

To identify novel modulators of autophagy among natural compounds, we screened an in-house library of 300 natural compounds derived from marine sponges, endophytic fungi, and higher plants, using a flow cytometric high-throughput screening (Fig. 1). The autophagic flux was measured in mouse embryonic fibroblasts (MEFs) expressing mCitrine-tagged LC3. As growth medium, we used serum-supplied full medium including amino acids, while for starvation, we used serum-free and amino acid-deficient starvation medium. During serum- and amino acid starvation, mCitrine-LC3 anchored to the inner autophagosomal membrane is degraded by lysosomal hydrolases, causing a reduction of the mCitrine-LC3 signal (Fig. 1A). Upon treatment with the lysosomal inhibitor bafilomycin A_1_, both basal and starvation-induced autophagic flux is inhibited and the mCitrine-LC3 signal increases (Fig. 1A), confirming the applicability of this assay to identify both inducers and inhibitors of autophagy.

For the classification of inducers and inhibitors, we set the threshold at a 15% decrease or increase of the mCitrine-LC3 signal upon treatment with the compounds. This threshold value is based on the positive control for autophagy induction in our cellular model system, i.e., Torin 2 treatment for 6 h. In addition, we set our threshold to compounds with a fairly moderate and higher effect according to statistical analyses of high-throughput data (│SSMD score│ ≥1) (Fig. 1, Tables 1 and 2, and Supplementary Table 1)^30^. In doing so, the screening of MEFs treated with natural compounds in full medium revealed five potential inhibitors of basal autophagy (Fig. 1B) and eight potential inducers of autophagy (Fig. 1B and Table 1). The screening in starvation medium revealed 64 potential inhibitors of autophagic flux (Fig. 1C and Table 2). In addition to the aforementioned five inhibitors, and four dual modulators (i.e., activating in full medium, but inhibitory under starvation), 55 compounds were classified as potential autophagy inhibitors. Of the 300 tested natural compounds, two-thirds did not have a considerable effect on autophagy (Fig. 1D).

**Table 1:** List of potential autophagy inducers.

Compound	Mean difference of mCit-LC3 (%)	SD	SSMD
P05E07	−34.4	±6.0	−3.25
P02E06	−28.8	±1.3	−12.58
P04C02	−27.4	±5.6	−2.75
P01F03	−22.0	±11.2	−1.11
P05C06	−19.9	±4.6	−2.43
P01G08	−18.1	±8.1	−1.26
P03F08	−17.7	±3.3	−3.04
P02B03	−17.3	±6.0	−1.62

*SD* standard deviation *SSMD* strictly standardized mean difference.

In total, eight compounds causing equal to or greater than 15% LC3 degradation at a fairly moderate and higher effect (│SSMD score│ ≥1) at 10 µM after 6 h of treatment were classified as potential inducers of autophagy.

Data show mean difference ± SD (% of control) of mCitrine-LC3 decrease upon full medium monitored by flow cytometry.

**Table 2:** List of potential autophagy inhibitors.

Compound	Mean difference of mCit-LC3 (%)	SD	SSMD
P05D05	+2590.4^a^	±765.1	1.91
P05C04	+536.3	±294.2	1.03
P02D03	+336.7	±182.2	1.04
P05D10	+335.7	±85.5	2.21
P04C10	+256.2	±45.0	3.21
P01B11	+249.9	±38.4	3.67
P03B08	+243.9	±45.4	3.03
P01F02	+240.8	±49.9	2.72
P03B06	+235.2	±32.0	4.14
P05B05	+234.4^a^	±44.8	2.95
P01E05	+224.6	±19.1	6.65
P05E10	+221.8	±21.1	5.92
P01C06	+220.6	±37.8	3.29
P01F11	+209.4	±55.6	2.13
P01G06	+174.5	±21.1	4.67
P04E03	+174.3	±30.7	3.21
P02D05	+170.9	±67.0	1.44
P04E04	+166.1	±48.5	1.93
**Arzanol**	+164.6	±63.9	1.45
P01F08	+159.6	±45.8	1.97
P05B09	+158.8	±53.8	1.67
P01G09	+158.7	±52.9	1.69
P04D02	+158.2	±19.3	4.63
P05B06	+153.1	±69.7	1.24
P05B02	+149.6	±47.0	1.80
P05C03	+149.0	±32.9	2.55
P03F09	+138.3	±37.5	2.08
P02E04	+122.0	±63.9	1.08
P02F04	+105.1	±55.0	1.08
P04G07	+98.3	±29.4	1.88
P02B07	+93.7	±44.5	1.19
P04B04	+93.4	±34.7	1.52
P04B07	+87.2	±39.5	1.24
P04F06	+78.1	±19.0	2.32
P05E07	+67.4	±19.5	1.95
P04B02	+67.1	±15.6	2.43
P04G08	+66.9	±30.5	1.24
P02B02	+60.7	±25.1	1.36
P05F02	+59.4	±12.7	2.64
P03F08	+53.3	±13.2	2.28
P05F03	+46.0	±14.1	1.84
P01C05	+41.8	±16.6	1.42
P04C02	+40.0	±16.0	1.41
P02E08	+38.7	±3.4	6.44
P05E06	+36.9	±15.9	1.31
P04E10	+35.4	±17.2	1.16
P05C06	+34.8	±5.1	3.85
P03E07	+33.6	±17.6	1.08
P05D06	+33.2	±12.4	1.51
P05G05	+33.2	±2.4	7.93
P02D07	+32.1	±17.3	1.05
P05E04	+31.3	±6.8	2.59
P01G02	+29.8	±14.2	1.18
P04C11	+27.6	±5.6	2.76
P01C09	+25.1	±6.8	2.10
P05F04	+23.8	±7.2	1.86
P04B10	+23.1	±8.3	1.57
P05D07	+22.9	±5.5	2.36
P01E03	+22.0	±9.0	1.39
P05E02	+21.1	±4.0	3.01
P04E02	+18.2	±9.2	1.12
P04D04	+17.2	±8.0	1.21
P05B10	+17.2	±7.5	1.30
P03E06	+15.9	±3.5	2.56

*SD* standard deviation, *SSMD* strictly standardized mean difference.

^a^Could be caused by autofluorescence.

In total, 64 compounds causing equal to or greater than 15% LC3 protection at a fairly moderate and higher effect (│SSMD score│ ≥1) at 10 µM after 6 h of treatment were classified as potential inhibitors of autophagy.

Data show mean difference ± SD (% of control) of mCitrine-LC3 increase upon starvation medium monitored by flow cytometry.

### Arzanol interferes with late-stage autophagy

**Fig. 2:**
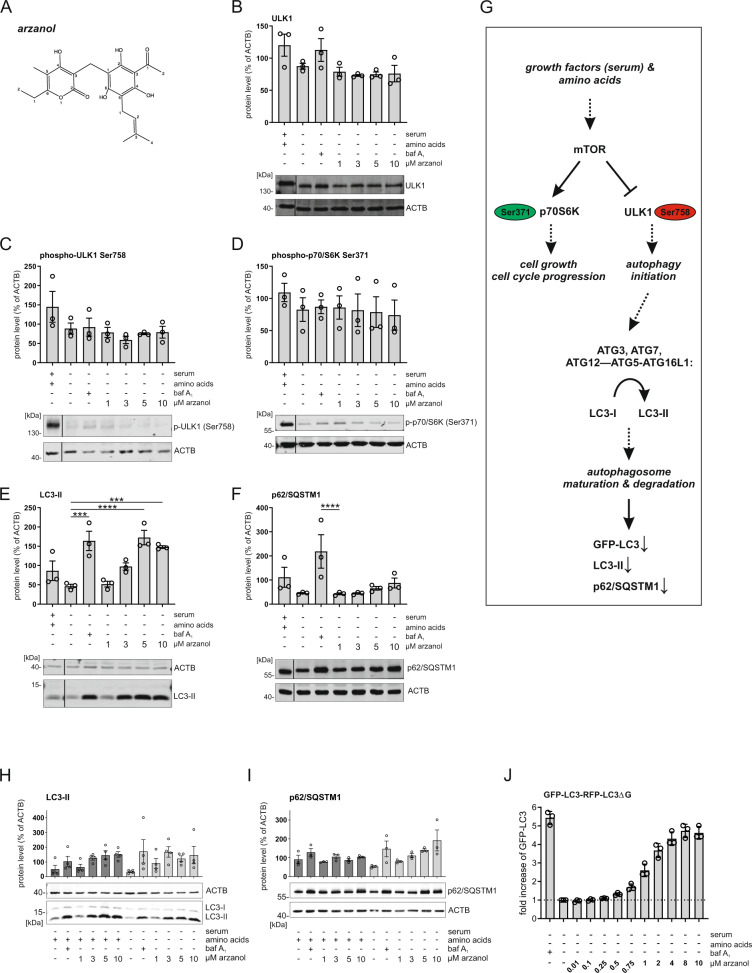
Arzanol drives LC3-II accumulation independent of the AKT/mTOR-pathway. **A** Structure of arzanol. **B**–**F** Immunoblot analysis of mTOR/ULK1-pathway proteins during serum- and amino acid starvation. Wild-type HeLa cells were starved in serum- and amino acid-free medium for 6 h while being incubated with baf A_1_ or different concentrations of self-isolated arzanol. Data show quantified means of biological triplicates normalized to β-actin (ACTB) ± SEM. Representative immunoblots are additionally shown. Nonadjacent lanes (i.e., full medium control lane) are indicated by vertical black lines. Statistical analysis was performed using two-way ANOVA with Dunnett’s multiple comparison test comparing treated to untreated samples, ***P < 0.001, ****P < 0.0001. **G** Simplified pathway of key proteins of the mTOR/ULK1-axis in starvation-induced autophagy. **H**, **I** Immunoblot analysis of LC3-II and p62/SQSTM1 during serum-starvation. Wild-type HeLa cells were starved in serum-free (dark bars) or serum- and amino acid-free (light bars) medium for 6 h while being incubated with baf A_1_ or different concentrations of commercially available arzanol. Data show quantified means of biological triplicates normalized to ACTB ± SEM. **J** HeLa cells stably expressing GFP-LC3-RFP-LC3ΔG were starved in serum- and amino acid-free medium for 6 h while incubated with different concentrations of commercially available arzanol. Data show fold increase of GFP-LC3 fluorescence relative to mock-treated control as means ± SEM of three biological replicates.

Among the novel modulators of autophagy, we identified the phloroglucinol α-pyrone arzanol (Fig. 2A; marked as red dots in Fig. 1B, C), which was isolated from the aerial parts of *Helichrysum italicum*^31^. We deliberately chose arzanol for further analysis because of the following reasons: (1) lack of significant cytotoxicity in the cell line used for screening, (2) identification of autophagy-modulating properties in orthogonal assays (e.g., LC3 turnover by immunoblotting, see below), (3) sufficient stability, (4) bioaccessibility, (5) price/commercial availability, and (6) lack of previous publications. Arzanol is known for its anti-inflammatory, antiviral^32^, antioxidative, and cytotoxic effects^33^, however, no effect on autophagy has been reported before. To identify the pathway affected by arzanol, we performed immunoblotting analyses of key components of the canonical autophagy pathway such as components of the mTOR-ULK1-axis (i.e., phosphorylation of ribosomal protein S6 kinase beta-1 (p70S6K) at Ser371 and ULK1 at Ser758), LC3, and p62/SQSTM1. During the early biogenesis of autophagosomes, cytosolic LC3-I gets lipidated to autophagosomal membrane-bound LC3-II, which is—along with the autophagy receptor p62/SQSTM1—later degraded by lysosomal hydrolases^34^.

We observed that arzanol does not inhibit starvation-induced inactivation of mTOR; however, it causes the accumulation of LC3-II and p62 during starvation-induced autophagy (Fig. 2B–F). We validated these effects using commercially available arzanol (Fig. 2H). The effect of arzanol on p62/SQSTM1 accumulation was more prominent upon amino acid starvation (Fig. 2I). We further validated the accumulation of LC3 in HeLa cells expressing the GFP-LC3-RFP-LC3ΔG reporter construct^35^ by flow cytometry, measuring the autophagic degradation of GFP-LC3 (Fig. 2J). The effect of arzanol on autophagy is consistent within all assays (i.e., murine vs. human cells, immunoblotting vs. flow cytometry, and self-isolated vs. commercial arzanol), which is why we used commercially available arzanol in all further experiments. Of note, we found that arzanol was ineffective in serum-supplied growth medium due to its serum-binding capacity (Supplementary Fig. 1A), which also explains the absence of effects in our screening in full medium (Fig. 1B). In serum-free and amino acid-rich medium, however, arzanol caused accumulation of LC3-II, which resembles its effects in the amino acid-free starvation medium (Fig. 2H). Furthermore, we exclude the involvement of antioxidative or scavenging effects of arzanol during amino acid starvation to be the reason for the observed effects, although antioxidative effects were observed in serum-free and amino acid-rich medium (Supplementary Fig. 1B).

### Arzanol changes ATG16L1 localization

**Fig. 3:**
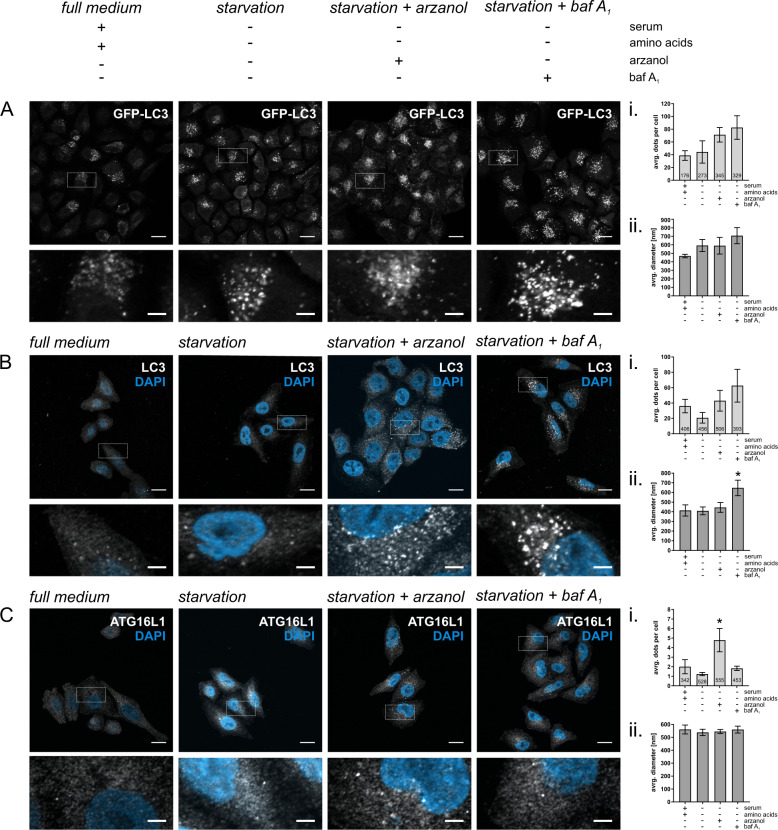
Arzanol interferes with autophagosome elongation. Shown are representative microscopy images of HeLa cells stably expressing GFP-LC3-RFP-LC3ΔG (**A**), or wild-type HeLa cells immunofluorescently labeled for endogenous LC3 (**B**) or ATG16L1 (**C**). All cells were starved in serum- and amino acid-free medium for 2 h while incubated with 3 µM arzanol or 10 nM bafilomycin A_1_. Scale bars in upper panels are 15.5 µm, scale bars in magnifications are 3.875 µm. (i) Data show the average number of dots per cell from biological triplicates for GFP-LC3, and *n* = 6 for LC3 and ATG16L1 as mean ± SEM. Digits in bars show the total number of cells quantified using ImageJ software. (ii) Data show the average diameter of dots in nm as mean ± SEM. Statistical analysis was performed using ordinary one-way ANOVA with Tukey’s multiple comparison test, *P < 0.1.

An increase in LC3 can be indicative of disrupted autophagic degradation or increased protein expression, while an increase in LC3-II can additionally hint to its enhanced lipidation. To determine the circumstances that led to the accumulation of LC3 and p62/SQSTM1 upon serum- and amino acid starvation (Fig. 2), we detected both overexpressed GFP-LC3 (Fig. 3A) and endogenous LC3 (Fig. 3B) using fluorescence microscopy and quantified both size and amount of LC3-positive dots that commonly represent autophagosomes^36^. A clear trend toward increasing numbers of LC3 dots upon arzanol treatment was observed (Fig. 3A, B). However, the size of these LC3 dots appeared rather small, making them partly indistinguishable for the quantification software. Nevertheless, we think one can appreciate the increase in the number of both overexpressed and endogenous LC3 dots and their significantly smaller size compared to bafilomycin A_1_-treated cells (Fig. 3A, B; i and ii.). Further, we examined the location, amount, and size of ATG16L1 dots, which is a core component of the LC3 lipidation machinery. Arzanol treatment caused a significant increase in the number of ATG16L1-positive structures (Fig. 3Ci), while the size of the dots and the total protein level of ATG16L1 remained unaltered (Fig. 3Cii and data not shown). Further, some of these ATG16L1 dots show a perinuclear localization, and are surrounded by a dense population of LC3-positive dots (Supplementary Fig. 2A). During autophagosomal membrane expansion and cargo sequestration, ATG16L1 is recruited to PE-containing phagophores by PI3P-binding WIPI2 (refs. ^37,38^). Of note, we did not observe an increased number of WIPI2 dots in response to arzanol (Supplementary Fig. 2B). Taken together, these data are in line with increased LC3-II in response to arzanol treatment we described above (Fig. 2E, H, J) and suggest an altered elongation process of autophagosomes upon arzanol treatment. Finally, we also investigated autophagic flux using the combination of arzanol with bafilomycin A_1_ (Supplementary Fig. 2C). We did not observe any differences between the combined treatment and the treatment with bafilomycin A_1_ alone, indicating that arzanol ultimately blocks autophagic flux.

### Arzanol as an anticancer drug in mono- and CDDP-combination therapy

**Fig. 4:**
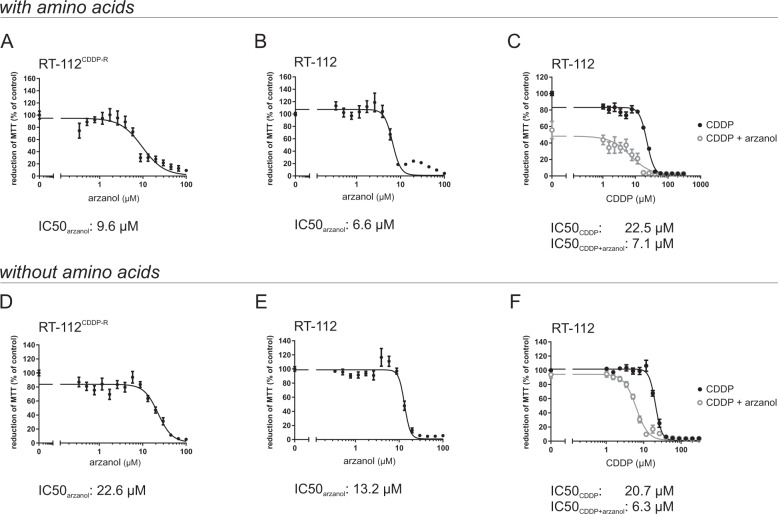
Arzanol is cytotoxic for bladder carcinoma cells in mono- and CDDP-combination therapy. Arzanol reduces cell viability in bladder cancer cells as determined by the MTT assay. CDDP-resistant (CDDP-R) RT-112 bladder carcinoma cells (**A**) and CDDP-sensitive RT-112 bladder carcinoma cells (**B**) were incubated for 24 h with indicated concentrations of arzanol in serum-free, amino acid-supplied medium. **C** CDDP-sensitive RT-112 bladder carcinoma cells were incubated with indicated concentrations of CDDP in monotherapy or in a combination with 5 µM arzanol in serum-free, amino acid-supplied medium. CDDP-resistant (CDDP-R) RT-112 bladder carcinoma cells (**D**) and CDDP-sensitive RT-112 bladder carcinoma cells (**E**) were incubated for 24 h with indicated concentrations of arzanol in serum- and amino acid-free medium. **F** CDDP-sensitive RT-112 bladder carcinoma cells were incubated with indicated concentrations of CDDP in monotherapy or in a combination with 5 µM arzanol in serum- and amino acid-free medium. The results are shown as mean ± SEM of at least three individual experiments performed in triplicates. All IC_50_ values were calculated using GraphPad Prism 7.01 (function log(inhibitor) vs. response – variable slope (four parameters)).

In addition to monotherapy, combinational therapies of approved drugs with autophagy modulators have been proposed to overcome tumor survival^39–41^. In order to test the cytotoxic effect of arzanol on cancer cells, we performed MTT assays after 24 h of either arzanol monotherapy or combinational therapy with cisplatin (CDDP) in RT-112 urothelial bladder carcinoma cells. We tested the cytotoxic effect of arzanol both in serum-free growth medium containing amino acids (Fig. 4A–C) and in serum- and amino acid-deficient starvation medium (Fig. 4D–F). The results of the cell viability assays reveal that arzanol is a moderate chemotherapeutic alone during amino acid supply in both CDDP-resistant cells (IC50: 9.6 µM; Fig. 4A) and in CDDP-sensitive RT-112 cells (IC50: 6.6 µM; Fig. 4B). For the amino acid-supplied CDDP-sensitive RT-112 cells, arzanol sensitizes the cells toward CDDP treatment (IC50 shift from 22.5 µM to 7.1 µM; Fig. 4C). Similarly, arzanol is also a moderate chemotherapeutic alone during amino acid starvation in both CDDP-resistant cells (IC50: 22.6 µM; Fig. 4D) and in CDDP-sensitive RT-112 cells (IC50: 13.2 µM; Fig. 4E). Thereby, arzanol appears less toxic during amino acid starvation in relation to the non-starved samples in both cell lines. For the amino acid-starved CDDP-sensitive RT-112 cells, arzanol still sensitizes the bladder cancer cells toward CDDP treatment (IC50 shift from 20.7 µM to 6.3 µM; Fig. 4F). In both nutrient conditions, arzanol did not sensitize the CDDP-resistant cell line (Supplementary Fig. 3A).

Surprisingly, when we compared these MTT assay data with data from an Alamar blue assay (Supplementary Fig. 3B), we noticed that especially for CDDP-resistant cells the treatment with low amounts of arzanol resulted in an increased reduction of MTT, but a decreased reduction of resazurin in the Alamar blue assay (Supplementary Fig. 3C). While both assays are colorimetric assays, the MTT assay measures the metabolic activity of enzymes that are capable to reduce the tetrazolium dye MTT 3-(4,5-dimethylthiazol-2-yl)-2,5-diphenyltetrazolium bromide to its insoluble formazan, whereas the Alamar blue assay depends on enzymes that reduce resazurin to resofurin. Similarly to our results, Hamid et al. observed the same phenomenon for the NAD(P)H quinone dehydrogenase 1 (NQO1) inhibitor dicoumarol^42^, which shares structural similarity with arzanol. In contrast to their observations on dicoumarol, we still witnessed that the cells die at higher concentrations of arzanol. Nevertheless, we suspect an effect of arzanol on the oxidoreductases involved in these assays.

### Arzanol targets mitochondria

**Fig. 5:**
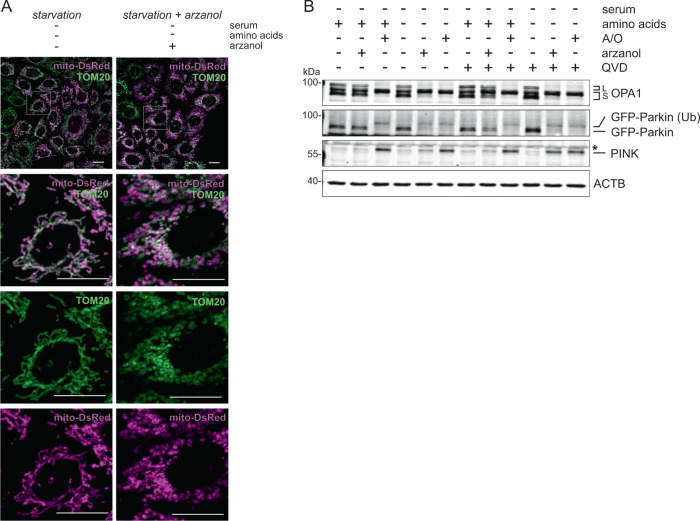
Arzanol causes damage-induced fragmentation of mitochondria during starvation. **A** Shown are microscopy images of mito-DsRed-expressing HeLa cells immunofluorescently labeled for endogenous TOM20. During starvation, arzanol induces fragmentation of both inner (mito-DsRed) and outer (TOM20) mitochondrial membranes. Cells were starved in serum- and amino acid-free medium for 2 h while incubated with 5 µM arzanol. Scale bars in all panels are 20 µm. **B** Immunoblot for markers of mitochondrial damage (OPA1 cleavage, Parkin ubiquitination (Ub), PINK accumulation) reveals that arzanol-induced mitochondrial fragmentation is independent of apoptosis induction. HeLa cells stably expressing mito-DsRed GFP-Parkin were incubated with serum-free full medium or starvation medium and treated with 5 µM arzanol or a combination of 4 µM antimycin and 10 µM oligomycin (A/O) with/without 10 µM QVD for 6 h. Shown blots are representative of three independent experiments. L and S indicate long and short forms of OPA1, respectively. Asterisk indicates an unspecific band.

In our search for upstream causes of ATG16L1 accumulation upon arzanol treatment, we next investigated mitochondrial integrity. Different mitotoxins stimulate mitophagy as a means of cell survival. They induce the fragmentation of damaged mitochondria as well as the recruitment of mitophagy-associated proteins PTEN-induced putative kinase protein 1 (PINK) and the E3 ubiquitin ligase Parkin^43–45^. In microscopy assays, arzanol induced the fragmentation of mitochondria determined with mito-DsRed localizing to the mitochondrial matrix and staining of the translocase of outer membrane 20 kDa subunit (TOM20) (Fig. 5A and Supplementary Fig. 4A). In line with this, arzanol caused the mitochondrial stress-induced cleavage of optic atrophy protein 1 (OPA1), presumable ubiquitination of Parkin, and stabilization of PINK primarily during amino acid starvation (Fig. 5B). Interestingly, the fragmented mitochondria seem adjacent to but not colocalized with the ATG16L1 dots observed upon arzanol treatment during serum- and amino acid starvation (Supplementary Fig. 4B).

**Fig. 6:**
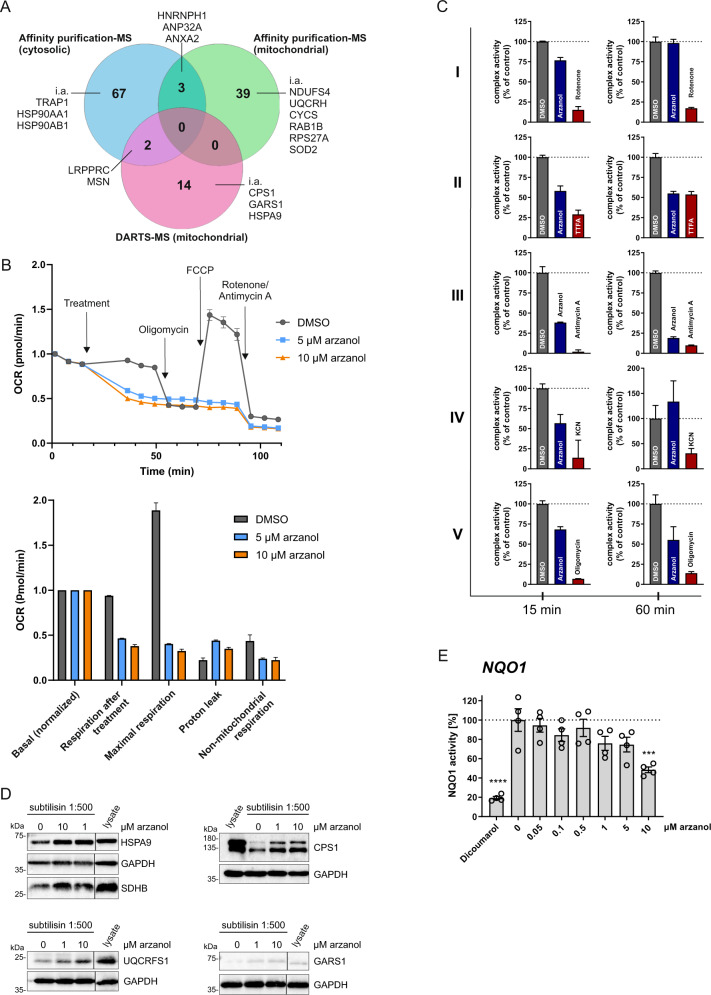
Target identification for arzanol reveals OXPHOS complexes and possibly the F_1_F_O_ ATP synthase/complex V as targets for arzanol. **A** Mass spectrometry (MS)-based target identification by affinity purification from HeLa cell whole lysates, and affinity purification and DARTS experiments from lysates of isolated mitochondria from HeLa cells reveal 125 novel potential targets of arzanol. **B** Cellular metabolism analysis was performed using a Seahorse Mito Stress Test Kit. Cells were incubated with arzanol for 15 min after measurement of the basal respiration and OCR was measured in at least octuplicates in three independent experiments. One representative experiment is shown (upper diagram). Respiration after treatment, maximal respiration, proton leak, and non-mitochondrial respiration were normalized to the basal respiration (lower diagram). Non-mitochondrial respiration = minimum rate measurement after rotenone/antimycin A injection; basal respiration = last rate measurement before first injection minus non-mitochondrial respiration; maximal respiration = maximum rate measurement after FCCP injection minus non-mitochondrial respiration; proton leak = minimum rate measurement after oligomycin injection minus non-mitochondrial respiration. **C** Arzanol inhibits mainly OXPHOS complexes II and III in a respiratory chain activity assay of isolated mitochondria after incubation for 15 min and 60 min. Complex activities were measured using the MitoCheck^®^ kits in technical triplicates and normalized to the DMSO control. Bars show mean + SEM. **D** DARTS experiment validates selected OXPHOS complex proteins of complexes II and III. The lysate of isolated mitochondria from HeLa cells was incubated with 1 or 10 µM arzanol and then subjected to subtilisin-dependent proteolytic cleavage. Arzanol stabilized SDHB, HSP9A, UQCRFS1, CPS1, and GARS1 against subtilisin treatment. Nonadjacent lanes (i.e., lysate control lane) are indicated by vertical black lines. Please note that in the SDHB/HSP9A panel the loading of the samples for 1 and 10 µM arzanol treatment are exchanged**. E** Arzanol reduces the enzyme activity in an NQO1 activity assay similar to the positive control 20 µM dicoumarol. Lysate from wild-type HeLa cells was subjected according to the manufacturer’s manual. Bars show mean ± SEM of biological quadruplets. Statistical analysis was performed using a one-way ANOVA with Dunnett’s multiple comparison test comparing treated to untreated control samples; ***P < 0.001, ****P < 0.0001.

Consistent with mitochondrial damage, we identified different mitochondrial proteins as novel protein targets for arzanol using two approaches. We performed affinity purification with arzanol covalently immobilized on beads and fished for binding-partners in HeLa and MEF cellular lysates and lysates from isolated mitochondria. We also carried out drug affinity responsive target stability (DARTS) assays in mitochondrial lysates to evaluate the enhanced stability of arzanol-binding targets against protease degradation. Both approaches were followed by mass spectrometry or immunoblotting to identify and confirm arzanol targets (Fig. 6A and Supplementary Table 2). Of note, we identified mitochondrial respiratory chain complex III proteins ubiquinol-cytochrome c reductase hinge protein (UQCRH) and cytochrome c somatic (CYCS), and complex I-related NADH:ubiquinone oxidoreductase subunit S4 (NDUFS4) bound to immobilized arzanol in affinity purification in mitochondrial lysates. We, therefore, monitored cellular oxygen consumption rates (OCR) in arzanol-treated and control cells using a Seahorse Extracellular Flux Analyzer (Fig. 6B). We observed that arzanol treatment reduced both the basal and the maximal OCR. Oligomcyin (inhibitor of F_1_F_O_ ATP synthase/complex V) did not result in a further reduction of the OCR and FCCP (uncoupler) did not stimultate cellular respiration, indicating that the oxidative phosphorylation (OXPHOS) complexes (complexes I–IV) and possibly also the F_1_F_O_ ATP synthase (complex V) are targeted by arzanol. We next tested arzanol directly in mitochondrial activity assays for OXPHOS complexes, and found that arzanol mostly inhibited complexes II and III, but also F_1_F_O_ ATP synthase/complex V (Fig. 6C). In line with these data, the succinate dehydrogenase complex iron sulfur subunit B (SDHB, OXPHOS complex II) and the ubiquinol-cytochrome c reductase Rieske iron–sulfur polypeptide 1 (UQCRFS1, OXPHOS complex III) were validated by immunoblotting assays of DARTS samples (Fig. 6D). Moreover, these immunoblotting assays also verified CPS1, GARS, and HSPA9 as arzanol targets. With the structural similarity of arzanol and dicoumarol, and the divergent effects of arzanol in MTT versus Alamar blue assays in mind, we also tested arzanol in a NQO1 activity assay. Arzanol reduced the activity of NQO1 in concentrations of 10 µM (Fig. 6E).

Taken together, our results show that arzanol sensitizes RT-112 bladder carcinoma cells towards CDDP, and reduces cell viability of both normal and CDDP-resistant RT-112 in monotherapy. Arzanol is able to target different quinone-dependent reductases such as NQO1 and oxidoreductases of the mitochondrial respiratory chain that maintain mitochondrial integrity. Arzanol reduces mitochondrial respiration and induces fission of mitochondria independent from reactive oxygen species. We observed the accumulation of ATG16L1 and lipidated LC3, but also smaller autophagosomal structures and the accumulation of p62/SQSTM1 upon arzanol treatment during starvation.

## Discussion

The modulation of autophagy is used as an anticancer treatment in more than eighty clinical trials treating several different cancer types (https://clinicaltrials.gov/; accessed on April 12, 2021). We used the top-down drug discovery approach to identify eight potential inducers and sixty-four potential inhibitors of autophagy. Among these compounds, we identified arzanol as a novel modulator of autophagy. Due to its strong effect on LC3-II accumulation and beneficial chemical characteristics like feasible compound isolation, commercial availability, and chemical stability, we selected it for a more detailed characterization.

In our assays, arzanol was initially identified as an inhibitor of starvation-induced autophagy by causing accumulation of overexpressed mCitrine-LC3 in murine fibroblasts. Consistently, it also caused the accumulation of overexpressed GFP-LC3 and endogenous total LC3 as well as lipidated LC3 in human cancer cells as determined by flow cytometry, fluorescence microscopy, and immunoblot analyses. Lipidation of LC3 is facilitated by the activation of the ubiquitin-like LC3 processing cascade involving ATG16L1^46^. Dudley et al. showed that ATG16L1 homodimerizes in order to bind to PI3P-covered pre-autophagosomal sites, and that modulating the PI3P-binding capacity of ATG16L1 impairs lipidation of LC3 (ref. ^12^). It is therefore not unlikely that the accumulation of ATG16L1 dots upon arzanol treatment under starvation conditions contributes to the increase in LC3-II observed in immunoblot analysis and to the increase in total LC3 measured by flow cytometry. As platforms for autophagosome biogenesis, ATG16L1-positive dots are also likely to account for the number of small LC3-positive dots detected by microscopy. However, also the inhibition of the autophagic flux likely contributes to these phenomena by preventing autophagosome maturation and lysosomal LC3 degradation. While we observed smaller autophagosomes upon arzanol treatment, it also caused accumulation of p62. We interpret the pronounced accumulation of p62 upon arzanol treatment during starvation as an indicator of an inhibited autophagic flux. This is clearly supported by our flux analysis using the combination of arzanol and bafilomycin A_1_. It remains unclear whether the dysregulation in LC3 lipidation itself is ultimately sufficient to inhibit autophagy or if additional targets are involved.

Bansal et al. observed that ATG16L1 is able to colocalize with LC3-binding mitophagy receptors such as ubiquitin-binding optic neuropathy inducing protein (optineurin/OPTN)^47–50^. Mitophagy is the selective removal of damaged mitochondria and aims to deplete damaged organelles and their associated triggers such as mitotoxins^43–45^. To induce mitophagy, PINK accumulates at the outer membrane of damaged mitochondria where it recruits and activates the E3 ubiquitin ligase Parkin, which then ubiquitinates mitochondrial surface proteins. This allows the recruitment of autophagic receptors such as NDP52 and OPTN, but also ULK1, FIP200, ATG9A, WIPI proteins, and ATG16L1 to form a mature autophagosome around the damaged organelle^51–53^. When we checked for mitochondrial damage induced by arzanol during serum- and amino acid starvation, we observed the fragmentation of the mitochondria. We further found accumulation of PINK in immunoblot analysis and observed GFP-Parkin to run at a higher molecular mass, which we interpret as ubiquitinated Parkin. Along with the markers for mitochondrial damage, we detected cleaved OPA1, a protein required for mitochondrial fusion^54–57^. In line with these data, we found arzanol to reduce the cellular OCR and the activity of mitochondrial OXPHOS complexes (mainly complexes II, III) and of complex V. Interestingly, among protein targets from affinity purification, we also identified NDUFS4, a protein of OXPHOS complex I. As novel targets for arzanol, we identified not only the complexes I, II, III and V, but also NQO1 and SOD2, which all function as quinone-dependent reductases required for mitochondrial integrity^58–62^. We hypothesize that arzanol binds to the quinone-binding pockets of these proteins and thus initiates the observed mitochondrial damage. TRAP1, another potential target of arzanol, regulates a metabolic switch between mitochondrial oxidative phosphorylation and glycolysis in cancer cells^63^. It is reported to maintain mitochondrial integrity downstream of OXPHOS complex I and PINK1^64^. We speculate that arzanol-induced mitochondrial damage provokes autophagosome formation and ATG16L1 accumulation.

We tested arzanol in monotherapy and combinational therapy with CDDP against sensitive and CDDP-resistant bladder cancer cells. CDDP-based chemotherapy is the first-line treatment in many cases of advanced or metastatic urothelial carcinoma^65^. However, innate and acquired chemoresistance remain a main reason for cancer-related lethality in patients. Similar to other autophagy inhibitors that reduced tumor growth rate and prolonged patient survival^66–70^, we found that monotherapy with arzanol reduced the viability of both CDDP-sensitive and -resistant bladder carcinoma cells while it sensitized the parental RT-112 cells to CDDP treatment in combination therapy. Clearly, in order to serve as lead compound for the development of anticancer drugs, the serum-binding properties of arzanol have to be addressed. We observed that arzanol was ineffective in the serum-supplied growth medium, thus preventing its systemic usage. Serum albumin has been suggested as a carrier for anticancer agents by prolonging the circulation half-life of drugs and thus promoting their accumulation within tumors^71^. Although our work represents a preclinical study based on cellular model systems, we hypothesize that it might be a worthwhile objective to derivatize arzanol in such a way that it keeps its serum-binding properties while regaining its biological activity.

Conclusively, from our data we characterize arzanol as an inducer of mitochondrial damage and autophagosome formation and as an inhibitor of autophagy. Its different targets might account for the multifaceted mode of action that leads to cytotoxic effects in bladder cancer cells. Taking into consideration its pharmacological activities, we propose arzanol as a new lead structure for the treatment of bladder cancer and suggest further investigations on its target- and cancer-specificity.

## Materials and methods

### Reagents

Natural compounds isolated from endophytic fungi, lichens, marine sponges, or plants were provided by Peter Proksch (Institute of Pharmaceutical Biology and Biotechnology of the Heinrich Heine University (Düsseldorf, Germany). Bafilomycin A_1_ (Sigma-Aldrich, #B1793) and arzanol (Sigma-Aldrich, #SBR00002) were dissolved in dimethyl sulfoxide (DMSO; AppliChem GmbH, #A3672). We purchased antimycin A from Sigma (#A8674) and oligomycin A from Toronto Research Chemicals (#O532970). For transfection of cells, we used FuGENE^®^ 6 (Promega, #E2692) and polybrene (hexadimethrine bromide; Sigma-Aldrich, #H9268-106). Immunoblots were performed using Immobilon™-FL PVDF membrane (Merck-Millipore, #IPFL00010), milk powder (Carl Roth, #T145.2), and Protease Inhibitor Cocktail powder (Sigma-Aldrich, #P2714-1BTL). Cells were cultivated using full medium DMEM (Gibco^®^ by Life Technologies, #41965-039), starvation medium EBSS (Gibco^®^ by Life Technologies, #24010-043), PBS (Gibco^®^ by Life Technologies, #14190-094), fetal bovine serum (GE Healthcare, #A15–101), 0.05% trypsin/EDTA solution (Gibco^®^ by Life Technologies, #25300-062), penicillin/streptomycin (10,000 U/ml, Biochrom GmbH, #A2213), puromycin (InvivoGen, ant-pr), or blasticidin (InvivoGen, ant-bl).

For immunoblotting, antibodies against ACTB/β-actin (Sigma-Aldrich, #A5316), LC3 (Cell Signaling Technology, #2775), SQSTM1/p62 (PROGEN Biotechnik, GP62-C), ULK1 (clone D8H5, Cell Signaling Technology, #8054), phospho ULK1 Ser758 (Cell Signaling Technology, #6888), phospho TSC2 Ser939 (Cell Signaling Technology, #3615), phospho p70S6K Ser371 (Cell Signaling Technology, #9208), OPA1 (described previously^72^), PINK (Cell Signaling Technology, #6946), Parkin (Abcam, #ab15954), SDHB (Thermo Fisher, #459230), UQCRFS1 (Invitrogen, #MA5-27471), CPS1 (Proteintech, #65011-1-Ig), GAPDH (Invitrogen, #437000), HSPA9/GRP 75 (clone D-9, Santa Cruz Biotechnology, #sc-133137) and GARS1/GlyRS (clone D-10, Santa Cruz Biotechnology, #sc-365311) were used. IRDye 800- or IRDye 680-conjugated secondary antibodies were purchased from LI-COR Biosciences (#926-32210/11, #926–68070/71, #926–68024, #926-68077, #926–32214).

For immunofluorescence, antibodies against ATG16L (MBL #PM040), LC3B (MBL #PM036 and #M152-3), WIPI2 (AbD Serotec/BIORAD, #MCA5780GA), TOM20 (Santa Cruz Biotechnology, #17764) were used. Alexa Fluor^®^ 488-conjugated and Alexa Fluor^®^ 647-conjugated antibodies were purchased from Jackson Immuno-Research Laboratories (Alexa Fluor^®^ 488 AffiniPure Donkey Anti-Mouse IgG (H + L), #715-545-151; Alexa Fluor^®^ 488 AffiniPure Goat Anti-Rabbit IgG (H + L), #111-545-003; Alexa Fluor^®^ 647 AffiniPure Goat Anti-Rabbit IgG (H + L), #111-605-003; Alexa Fluor^®^ 647 AffiniPure Goat Anti-Mouse IgG (H + L), #115-605-003).

NQO1 activity of HeLa cells was measured using the NQO1 Activity Assay Kit (Abcam, #184867) according to the manufacturer. OXPHOS complex activities were measured from isolated mitochondria using the MitoCheck Complex Activity Assay Kits (Cayman Chemicals, #700930, #700940, #700950, #700990, #701000). Both assays were measured using a microplate reader (BioTek, Synergy Mx). Cellular metabolism analysis was carried out using a Mito Stress Test Kit (Agilent Technologies, # 103015-100); this kit includes oligomycin, FCCP, rotenone, and antimycin A.

### Generation and culture of cell lines

Wild-type mouse embryonic fibroblasts (MEFs) (kindly provided by Tullia Lindsten, Memorial Sloan Kettering Cancer Center, New York City, USA) were retrovirally transfected with pMSCVblast/mCitrine-LC3B. Generation of pMSCVblast/mCitrine-LC3B was described previously^73^. For transfection, Plat-E cells (kindly provided by Toshio Kitamura, Institute of Medical Science, University of Tokyo, Japan) were transfected with 1.9 μg pMSCV-based retroviral vectors using FuGENE^®^ 6 (Promega) transfection reagent according to the manufacturer’s manual. After 48 h, retroviral supernatant was collected and used for the infection of MEFs in combination with 9 µg/mL polybrene (Sigma-Aldrich; H9268-106). The cells were incubated for 3 days prior to selection with 35 μg/ml blasticidin.

Wild-type HeLa cells (kindly provided by Richard Youle, John Edward Porter Neuroscience Research Center, Bethesda, USA) were retrovirally transfected with pMRX-IP/GFP-LC3-RFP-LC3ΔG (kindly provided by Noboru Mizushima, Department of Biochemistry and Molecular Biology, University of Tokyo, Tokyo, Japan; Addgene plasmid #84572; http://n2t.net/addgene:84572; RRID:Addgene_84572). Alternatively, mito-DsRed-expressing HeLa cells (kindly provided by Aviva M. Tolkovsky, Department of Clinical Neurosciences, University of Cambridge, Cambridge, United Kingdom) were retrovirally transfected with pMSCVpuro/EGFP-Parkin. This plasmid was generated by inserting EGFP cDNA and human Parkin cDNA into pMSCVpuro. For retroviral transfection of HeLa cells, Plat-E cells were transfected with 1.9 μg pMRX- or pMSCVpuro-based retroviral vectors and 1.0 µg pVSV-G vector DNA using FuGENE^®^ 6 (Promega) transfection reagent according to the manufacturer’s manual. After 48 h, retroviral supernatant was collected and used for the infection of HeLa cells in combination with 9 µg/mL polybrene (Sigma-Aldrich; H9268-106). The cells were incubated for three days prior to selection with 2.5 μg/ml puromycin. For Fig. 2J, cells were subcloned in order to isolate clones that properly express GFP-LC3-RFP-LC3ΔG, since homologous recombination can occur between the two LC3 sequences during transfection^35^.

All cell lines including urothelial bladder carcinoma cells RT-112 and their CDDP-resistant equivalent cell line (previously described^74^ and kindly provided by Margaretha Skowron, Department of Urology, Medical Faculty, Heinrich Heine University, Düsseldorf, Germany) were cultured in high glucose (4.5 g/l) DMEM supplemented with 10% FCS at 37 °C in a 5% CO_2_ humidified atmosphere. For CDDP-resistance, RT-112 cells were kept at 12 µg/ml CDDP. For amino acid starvation, cells were washed once with PBS and incubated for the indicated times in EBSS.

### High-throughput autophagy screening

Mouse embryonic fibroblasts stably expressing mCitrine-LC3 were incubated with 10 µM of each natural compound solved in DMSO in serum-containing full medium or starvation medium for 6 h. During screening, compound samples were blinded by labeling with a randomized code and unblinded post-experiments. Cells were harvested by trypsinization, washed using PBS and centrifugation at 300 × *g*, and measured via flow cytometry of 10,000 events in the FITC channel of an LSRFortessa (Becton Dickinson, Heidelberg, Germany). Median fluorescence intensities were measured in biological triplicates and normalized to the DMSO control.

### GFP-LC3-RFP-LC3ΔG assay

HeLa cells stably expressing GFP-LC3-RFP-LC3ΔG were incubated with the indicated concentrations of self-isolated or commercial arzanol solved in DMSO in the starvation medium for 6 h. Cells were harvested and analyzed using flow cytometry as described above.

### Immunoblotting

Cleared cell lysates were prepared and subjected to immunoblotting as described before^73^. Signal intensities of protein bands were quantified using Image Studio Lite 4.0 (LI-COR), and each band was normalized to the average protein signal to correct for technical variance. The signals were then normalized to the corresponding loading control (ACTB). Panels for at least three biological replicates were prepared using GraphPad Prism 7.0.

### Fluorescence microscopy

On the day before treatment, cells were grown on glass coverslips (Marienfeld). After treatment, cells were fixed with 4% formaldehyde-PBS for 30 min on ice and quenched with 50 mM NH_4_Cl for 15 min. For immunofluorescence labeling, cells were then permeabilized with 0.2% Triton X-100-PBS for 15 min, or 50 µg/ml digitonin (Roth, #4005) for 5 min according to the antibody manufacturers. Samples were blocked with 3% BSA (Roth, #8076)-PBS for 30 min and incubated with primary antibodies for 1–2 h. After washing and 30 min of secondary antibody incubation, samples were again washed three times with PBS. Cells were embedded in ProLong Glass Antifade Mountant (Thermo Fisher Scientific, #P36980), including DAPI. Imaging was performed with a Zeiss Axio Observer 7 fluorescence microscope (Zeiss, Köln, Germany) with a Plan Apochromat 40x/1.4 oil objective (Zeiss, Köln, Germany). Quantification of images was performed with ImageJ. For that, signals and nuclei were counted per image and a signal-to-nuclei ratio was calculated. Macros for the quantifications are provided in Supplementary Methods.

### Cell viability assays

Cell viability was determined using the colorimetric Alamar Blue and MTT assays, which measure the reduction of non-fluorescent dyes to the fluorescent metabolites resorufin and formazan, respectively. For both assays, cells were cultivated in 96-well plates. The following day, the cells were treated with cisplatin and/or arzanol for 24 h. For the MTT assay, MTT (Roth #4022) was added to the cells at 0.5 mg/ml concentration and incubated at 37 °C for 1 h. Afterward, the plates were centrifuged at 600 × *g* and 4 °C for 5 min, and cells were lysed in DMSO for 20 min in the dark. Finally, the absorbance was measured at 570 nm and 650 nm for reference, using a microplate reader (BioTek, Synergy Mx). For the Alamar blue assay, 40 µM resazurin sodium salt (Cayman Chemicals, #14322) was added to the cells and incubated at 37 °C for 3 h. Afterward, the absorbance was measured at 590 nm, using a microplate reader (BioTek, Synergy Mx). The mean of the absorbance of untreated control samples was set as 100%.

### Cellular metabolism analysis

For cellular metabolism analysis, a Mito Stress Test Kit (Agilent Technologies, Santa Clara, CA, USA) was applied according to the manufacturer’s instructions using a Seahorse XFe96 Extracellular Flux Analyzer (Agilent Technologies). The FCCP concentration of 2.5 µM and the cell density of 15,000 cells/well were titrated prior to the experiments. Treatment with arzanol was performed after measurement of basal respiration. Basal respiration can be defined as respiration before the first injection by the Seahorse system. The maximal respiration was defined as the OCR after FCCP injection minus the OCR after blocking the mitochondrial respiration after rotenone and antimycin A injection (non-mitochondrial OCR). Proton leak is defined as the minimum rate measurement after oligomycin injection minus the non-mitochondrial respiration.

### Isolation of mitochondria

For isolation of mitochondria, wild-type HeLa cells were cultivated on 150-mm diameter tissue culture-treated dishes (Sarstedt) and ~3.6 × 10^8^ cells were harvested the next day via scraping. Cells were pelleted at 500 × *g* for 5 min and washed twice with PBS (Gibco). The pellet was resuspended in 10 ml mitochondria isolation buffer (210 mM mannitol, 70 mM sucrose, 1 mM EDTA, 20 mM HEPES, and protease inhibitor cocktail (Sigma-Aldrich, #P2714)) for 5 min on ice before rupturing by seven strokes through a 26 G cannula. The cell lysate was then centrifuged at 1000 × *g* and 4 °C for 5 min and the supernatant was collected. The remaining pellet of non-lysed cells was resuspended in 2 ml of mitochondria isolation buffer and ruptured again before centrifugation, and the two fractions were pooled. The pooled lysate was centrifuged again at 1000 × *g* and 4 °C for 5 min, and the pellet was discarded. The remaining lysate was centrifuged at 8000 × *g* and 4 °C for 10 min. The supernatant (cytosolic fraction) was collected and centrifuged again before transferring into a new tube and freezing in liquid nitrogen. The pellet (mitochondrial fraction) was washed three times at 8000 × *g* for 10 min in 250 µl of mitochondria isolation buffer. The pellet was finally centrifuged at 10,000 × *g* and 4 °C for 10 min. The supernatant was discarded and the pellet containing isolated mitochondria was frozen in liquid nitrogen and stored at −80 °C.

### Preparation of cellular and mitochondrial HeLa lysates

Cellular HeLa lysates were obtained by mechanical lysis in PBS pH 7.4 (137 mM NaCl, 2,7 mM KCl, 10 mM Na_2_HPO_4_, 2 mM KH_2_PO_4_) containing 0.1% IGEPAL CA-630 and a protease inhibitor cocktail (Sigma-Aldrich). Mitochondria were lysed in a buffer composed of 1.5% digitonin, a protease inhibitor cocktail (Sigma-Aldrich), 150 mM NaCl, 10 mM Tris/HCl (pH 7.5), and 5 mM EDTA. After 15 min at 4 °C, debris was discarded by centrifugation at 20,000 × *g* (30 min at 4 °C). The protein concentration of the obtained supernatants was determined by Bradford spectrophotometric assay (BioRad Laboratories, Hercules, CA) and adjusted to 3 mg/ml.

### Affinity purification from lysates and isolated mitochondria

Arzanol bearing resin has been obtained as reported by Del Gaudio et al.^75^. The arzanol-containing and the control resin were separately incubated with a solution containing 800 µg of proteins for 1 h under stirring at 4 °C. After the incubation period, unspecifically adsorbed proteins were removed performing three rounds of washing with PBS, whereas bound proteins were eluted with 50 μl of 100 mM Tris (pH 6.8), 4% (v/v) sodium dodecyl sulfate (SDS), 0.2% (v/v) Blue Bromophenol, 20% (v/v) glycerol, and 2% β-mercaptoethanol buffer. Each resin was then boiled at 95 °C for 5 min and 15 μl of the obtained eluates were subjected to 1D-SDS-PAGE (12% polyacrylamide). The resulting gel was then treated with 40% MeOH, 10% CH_3_COOH, and 50% H_2_O and stained by Coomassie Blue.

### Drug affinity responsive target stability

In total, 300 μg protein aliquots were either incubated with DMSO (vehicle control) or with arzanol (1 and 10 μM final concentrations) for 1 h at room temperature and under agitation. The obtained samples were then treated with the unspecific protease subtilisin (Sigma-Aldrich, #P5380) (enzyme to proteins ratio of 1:500 w/w) and left shaking for 30 min at 25 °C. Proteolysis was then quenched by adding PMSF (phenylmethylsulfonyl fluo-ride, Sigma-Aldrich, #P7626, 1 mM final concentration) to each sample. Subsequently, all of the samples were prepared for 1D-SDS-PAGE and 20 μg were loaded on a 4–12% Bis-Tris Criterion^TM^ XT Precast Gel (BioRad Laboratories, #3450123), which was then stained with a Coomassie solution. The experiment was carried out in duplicates.

### In situ gel digestion

Protein bands were excised from the gels and submitted to an in situ tryptic digestion protocol^76^. Briefly, gel slices were reduced by 6.5 mM 1,4-dithiothreitol (DTT) and alkylated by 54 mM iodoacetamide. A 12 ng/µl trypsin/LysC solution (Promega, Madison, Wisconsin) was used to digest proteins. The enzyme excess was then discarded and replaced with ammonium bicarbonate (AmBic, 40 µl, 50 mM, pH 8.5), allowing protein digestion to carry on overnight at 37 °C. Subsequently, supernatants were collected and peptides were extracted from each gel slice using 100% CH_3_CN. The obtained peptide mixtures were dried in vacuo and dissolved in formic acid (FA, 10%) for LC-MS/MS analysis.

### Liquid chromatography and mass spectrometry analysis (LC-MS/MS)

In total, 5 µl of each sample were injected into a nano-ACQUITY UPLC system (Waters, Milford, MA, USA), equipped with a 1.7-µm BEH C18 column (Waters). Peptide elution was achieved with a linear gradient of mobile phase B from 20 to 90% in 65 min (mobile phase A: 95% H_2_O, 5% CH_3_CN, 0.1% acetic acid; mobile phase B: 95% CH_3_CN, 5% H_2_O, 0.1% acetic acid) at a flow rate of 280 nl/min. MS and MS/MS data were acquired on an LTQ Orbitrap XL high-performance liquid chromatography MS system (Thermo-Scientific, Waltham, MA, USA), interfaced with a nanoESI source. The ten most intense doubly and triply charged peptide ions were fragmented. MS data were then processed by the MS Converter General User Interface software (ProteoWizard; http://proteowizard.sourceforge.net/project.html) and submitted to MASCOT Deamon (version 5.1, Matrix Science, London, UK) for protein identification, employing the SwissProt database (release November 2019, 561,344 entries) and the following settings: two missed cleavages; carbamidomethyl (C) as fixed modification; oxidation (M) and phosphorylation (ST) as variable modifications; peptide tolerance 30 ppm; MS/MS tolerance 0.8 Da.

### Statistics

Sample sizes were chosen with reference to pragmatic considerations and the principle of saturation due to previously published work in this field and preliminary experiments. For high-throughput analysis of the flow cytometric mCitrine-LC3-based autophagy screening, we used uniformly minimal variance unbiased estimate (UMVUE) of strictly standardized mean difference (SSMD)^30^. For the quantifications of the immunoblots shown in Fig. 2, statistical analysis was performed using a two-way ANOVA with Dunnett’s multiple comparison test comparing treated to untreated control samples. For comparing size and number of dots in Fig. 3, statistical analysis was performed using an ordinary 1way ANOVA with Tukey’s multiple comparison test comparing all treatments with each other. All IC_50_ values in Fig. 4 were calculated using GraphPad Prism 7.01 (function log(inhibitor) vs. response − variable slope (four parameters)). For NQO1 activity data (Fig. 6E), statistical analysis was performed using a one-way ANOVA with Dunnett’s multiple comparison test comparing treated to untreated control samples. Representative data or means of biological replicates are shown for every experiment with error bars that indicate standard error. Numbers of technical or biological replicates and definition of *P* values are indicated in the corresponding figure legend. All statistical analyses were performed using Prism v7.01 (GraphPad Software, La Jolla, CA, USA).

## Supplementary information

Supplementary Figures

Supplementary Methods

Supplementary Table 1

Supplementary Table 2

## Data Availability

The datasets used and/or analyzed during this study are available from the corresponding author on reasonable request.
