# Supplementary material for: High-throughput screening for natural compound-based autophagy modulators reveals novel chemotherapeutic mode of action for arzanol
Source: Cell Death Dis. 2021 May 31;12(6):560. doi: 10.1038/s41419-021-03830-5 (PMC8167120; doi:10.1038/s41419-021-03830-5)
Supplement: Supplementary file 1 — Supplementary Figures [file 41419_2021_3830_MOESM1_ESM.pdf]

High-throughput screening for natural compound-based autophagy modulators reveals novel chemotherapeutic mode of action for arzanol

Jana Deitersen[1], Lena Berning[1], Fabian Stuhldreier[1], Sara Ceccacci[2], David Schlütermann[1], Annabelle Friedrich[1], Wenxian Wu[1], Yadong Sun[1], Niklas Berleth[1], María José Mendiburo[1], Sabine Seggewiß[1], Ruchika Anand[3], Andreas S. Reichert[3], Maria Chiara Monti[2], Peter Proksch[4], Björn Stork[1,*]

[1]Institute of Molecular Medicine I, Medical Faculty and University Hospital Düsseldorf, Heinrich-Heine-University Düsseldorf, Universitätsstraße 1, 40225 Düsseldorf, Germany

[2]Department of Pharmacy, University of Salerno, Via Giovanni Paolo II 132, 84084 Fisciano Salerno, Italy

[3]Institute of Biochemistry and Molecular Biology I, Medical Faculty and University Hospital Düsseldorf, Heinrich-Heine-University Düsseldorf, Universitätsstraße 1, 40225 Düsseldorf, Germany

[4]Institute of Pharmaceutical Biology and Biotechnology, Faculty of Mathematics and Natural Sciences, Heinrich-Heine-University Düsseldorf, Universitätsstraße 1, 40225 Düsseldorf, Germany

Legends to Supplementary Tables 1 & 2

Supplementary Figures 1-4

Table S1:

Raw data of analyses shown in figures 1B-D.

Table S2:

Arzanol putative targets identified by AP-MS, DARTS-MS and enzymatic assays are reported. AP-MS identified targets are described within the following parameters: molecular weight (mass), mascot Score (Score), number of matched peptides (Matches) and unique peptides used in the identification process (Match(sig)), number of sequences (Sequences) and the number of significant distinct sequence matches in the protein identification process (Seq(sig)) and the relative quantitation of each protein (emPAI). The targets are grouped in protein families and only the families with at least two members are reported both from human and/or mouse cells. DARTS identified targets are reported together with their molecular weight (mass) and the number of matched peptides (Matches) for each experiment of the duplicate at different concentrations of arzanol. Higher are the matches, more protected is the protein.

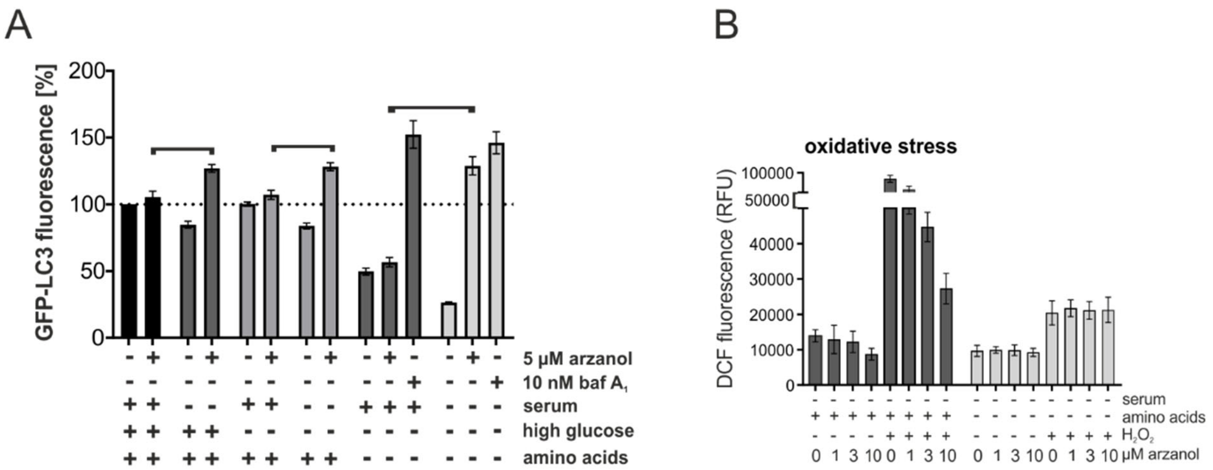

Figure S1 (related to figure 2): Serum-binding, non-scavenging/non-oxidative, and anti-autophagic properties of arzanol. (**A**) The addition of serum to medium attenuates the effect of arzanol treatment on LC3 accumulation in HeLa cells. HeLa cells stably expressing GFP-LC3-RFP-LC3ΔG were incubated with arzanol in different media containing high or low glucose concentrations, with/without serum, with/without amino acid supplementation. Data were normalized to full medium (high glucose incl. FCS, and DMSO) mock control and are shown as mean ± SEM, n=3. (**B**) Arzanol does neither increase nor reduce the oxidative stress measured in HeLa cells in amino acid-free starvation medium after 1 h. Wild-type HeLa cells were pre-incubated with 20 µM H2DCF-DA at 37°C for 30 min prior to washing steps and incubation with 1 M H_2O_2 and/or 1, 3 or 10 µM commercially supplied arzanol. DCF fluorescence was measured in a BioTek Synergy MX Microplate Reader at Em495/Ex527 and 37°C for 1 h. Data are shown as mean ± SEM, n=3.

S2 (Supplement to figure 3): Unaltered WIPI2 signal upon arzanol treatment.

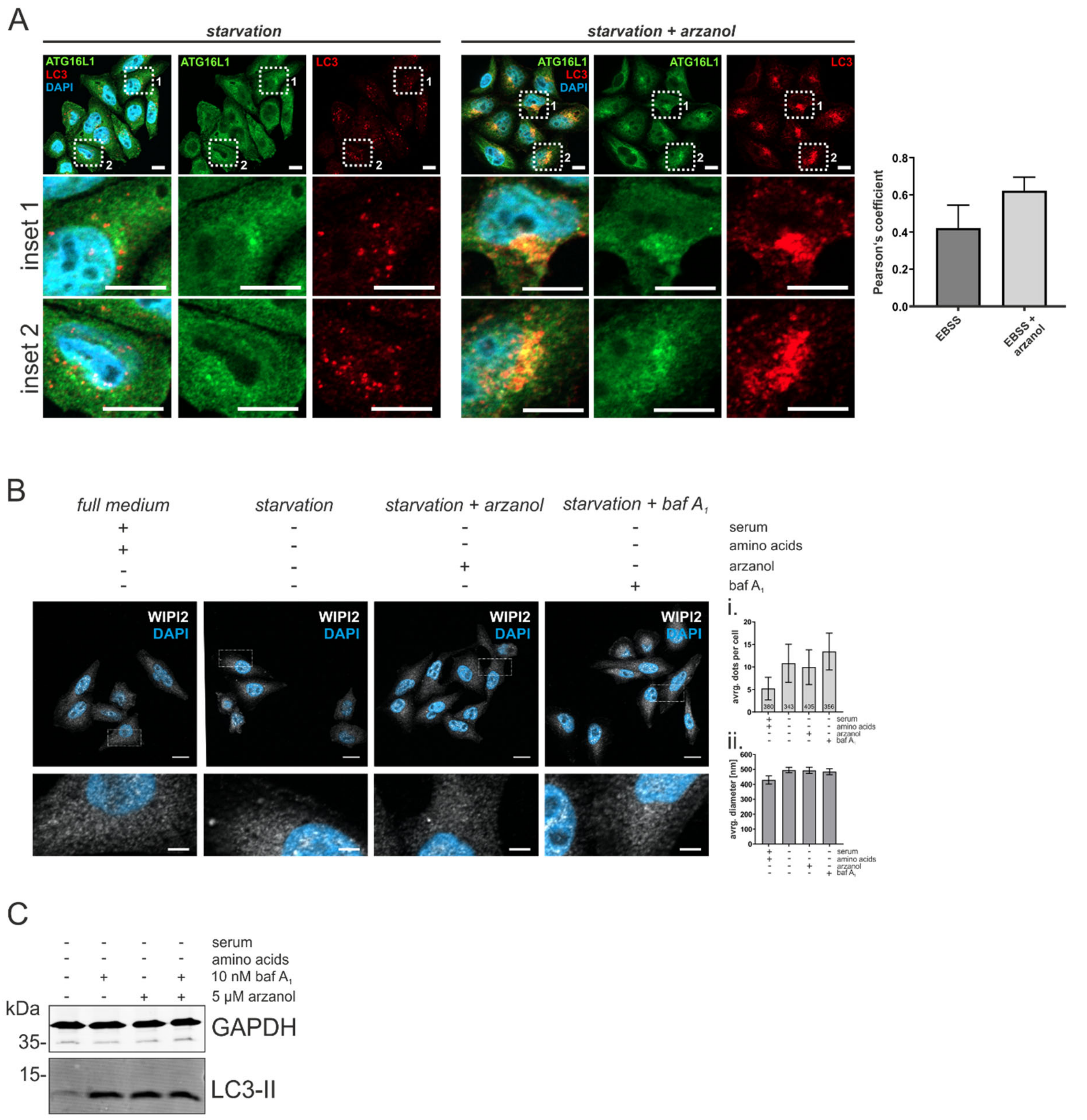

Figure S2 (related to Figure 3): Arzanol induces colocalization of ATG16L1 and LC3 and inhibits autophagic flux, but does not alter WIPI2 localization. (**A**) Shown are representative microscopy images of wild-type HeLa cells immunofluorescently labelled for endogenous ATG16L1 and LC3; nuclei were stained with DAPI. Cells were starved in serum- and amino acid-free medium without or with 3 µM arzanol for 2 h. Scale bars in panels are 10 µm. The colocalization intensity was analyzed using Pearson's correlation coefficient after Costes automatic thresholding using ImageJ software. At least 9 images from 3 biological replicates were analyzed for each treatment. (**B**) Shown are representative microscopy images of wild-type HeLa cells immunofluorescently labelled for endogenous WIPI2. Cells were grown in full medium or starved in serum- and amino acid-free medium for 2 h while incubated with 3

μM arzanol or 10 nM bafilomycin A_1. Scale bars in upper panels are 15.5 μm, scale bars in magnifications are 3.875 μm. (i.) Data show average number of dots per cell as mean ± SEM (n=5). Digits in bars show total number of cells quantified using ImageJ software. (ii.) Data show average diameter of dots in nm as mean ± SEM. Statistical analysis was performed using ordinary oneway ANOVA with Tukey's multiple comparison test. (**C**) Arzanol does not further increase LC3-II accumulation in combination with bafilomycin A_1. LC3 lipidation detected by immunoblot of wild-type HeLa cells treated with 5 μM commercial arzanol and/or 10 nM of bafilomycin A_1 in starvation medium for 4 h.

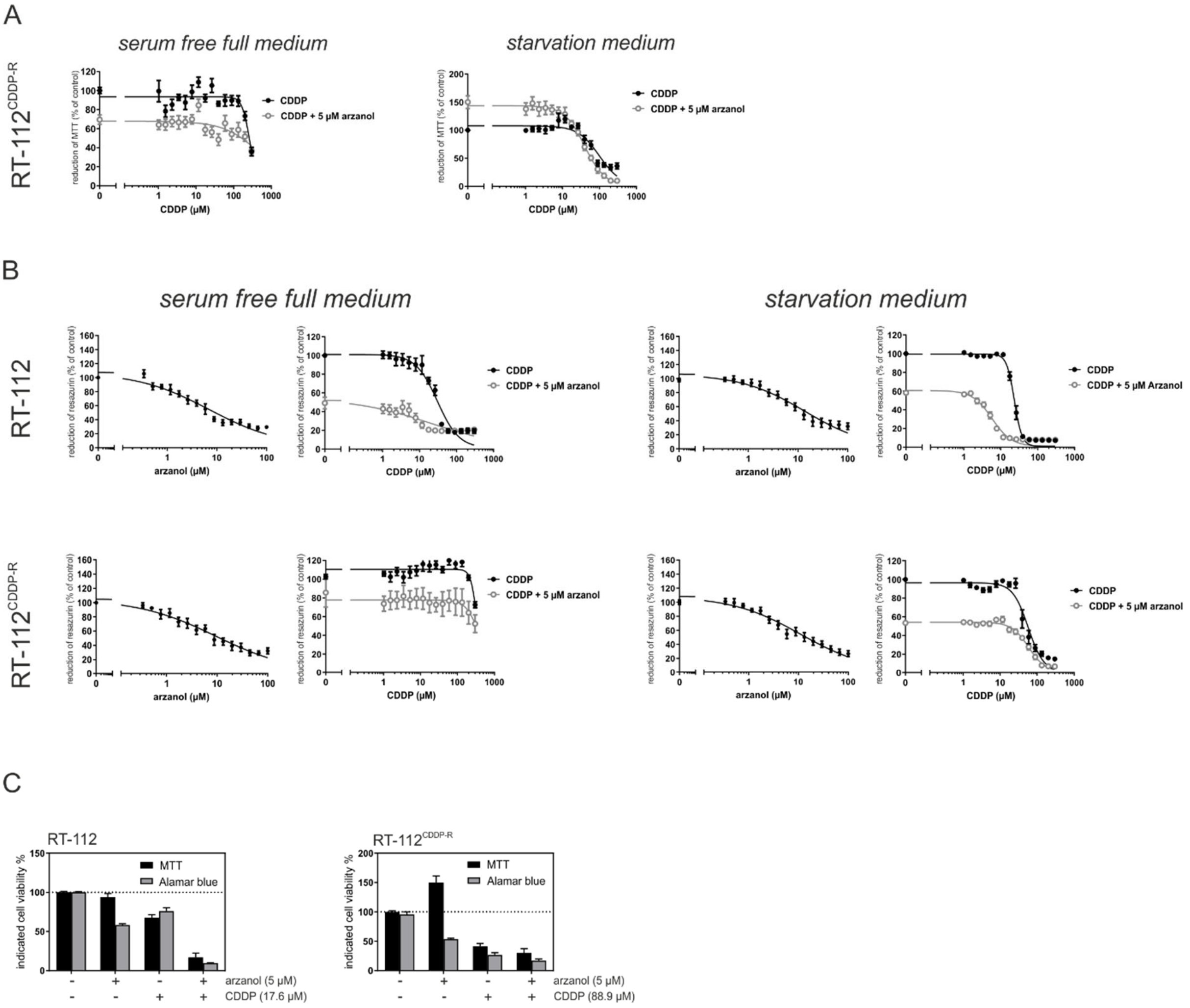

Figure S3 (related to Figure 4): Effect of arzanol in bladder carcinoma cells. (**A**) CDDP-resistant RT-112 bladder carcinoma cells were incubated with indicated concentrations of arzanol or indicated concentrations of CDDP ± 5 µM arzanol during starvation for 24 h. Cell viability was measured using an MTT assay. (**B**) CDDP-sensitive and -resistant RT-112 bladder carcinoma cells were incubated with indicated concentrations of arzanol or indicated concentrations of CDDP ± 5 µM arzanol during starvation or upon serum free full medium for 24 h. Cell viability was measured using an Alamar blue assay. (**C**) The left diagram shows a depiction of the synergistic effect of 5 µM arzanol and 17.6 µM CDDP comparing MTT and Alamar blue assay in starved CDDP-sensitive RT-112. The right diagram shows a depiction of the synergistic effect of 5 µM arzanol and 88.9 µM CDDP comparing MTT and Alamar blue assay in starved CDDP-resistant RT-112. The results are shown as mean ± SEM of four individual experiments performed in triplicates for treatments with arzanol alone, and six biological replicates performed in technical triplicates for combination of arzanol and CDDP.

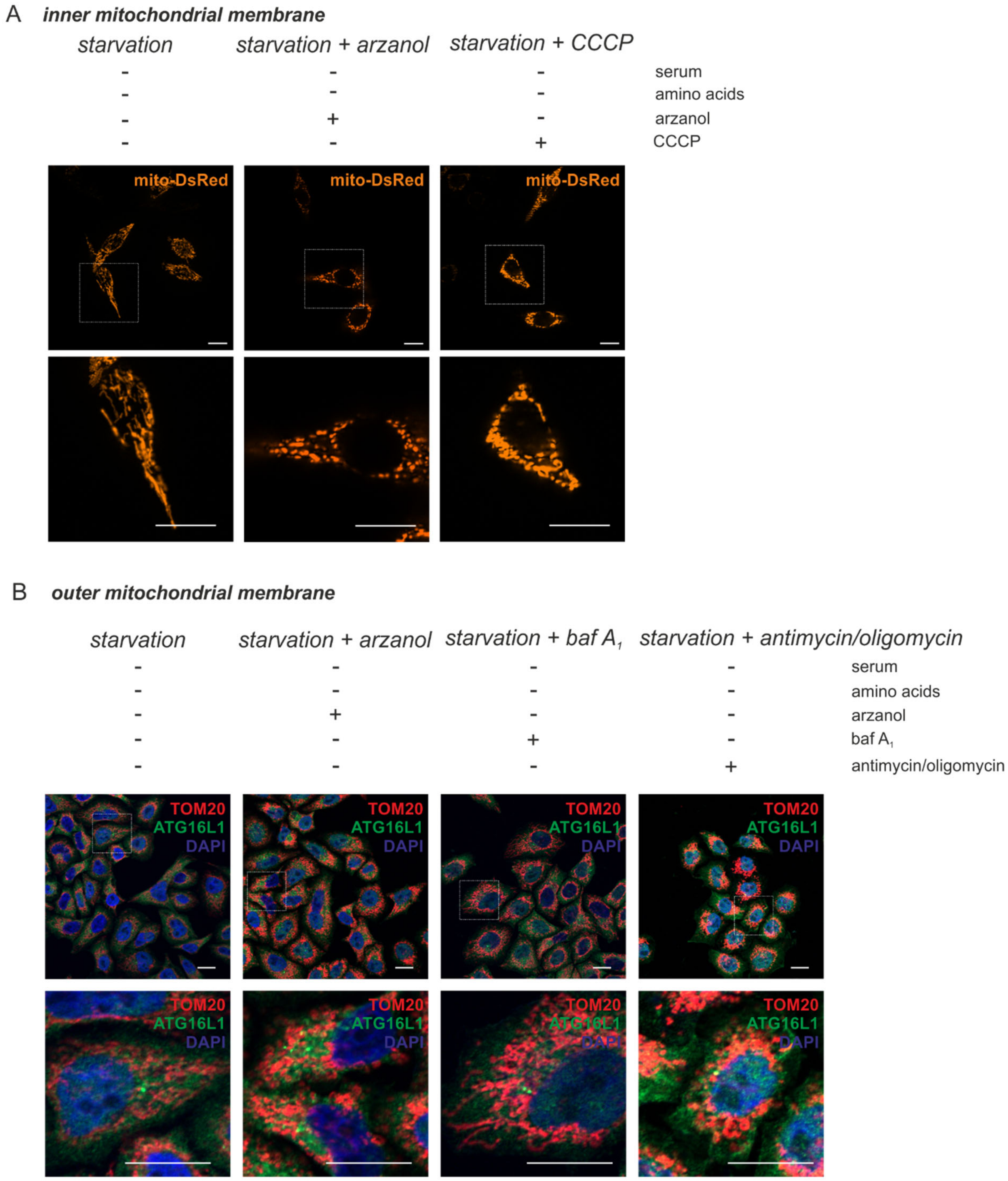

Figure S4 (related to figure 5): Fragmentation of mitochondria upon arzanol in starvation medium. (**A**) Shown are microscopy images of mito-DsRed-expressing HeLa cells. Upon starvation, arzanol induces a fragmentation of mitochondria. Cells were starved in serum- and amino acid-free medium for 15 min while incubated with 5 µM arzanol. 500 µM CCCP (mitochondrial encoupler) served as positive control. (**B**) Shown are microscopy images of wild-type HeLa cells. Upon starvation, arzanol induces a fragmentation of mitochondria stained for TOM20. Cells were starved in serum- and amino acid-free medium for 2 h while incubated with 5 µM arzanol or 10 nM bafiolomycin A_1. A combination of 4 µM antimycin and 10 µM oligomycin served as positive control for mitochondrial damage. Scale bar in panels are 15.5 µm, scale bars in magnifications are 3.875 µm.
