# Supplementary material for: High-throughput screening for natural compound-based autophagy modulators reveals novel chemotherapeutic mode of action for arzanol
Source: Cell Death Dis. 2021 May 31;12(6):560. doi: 10.1038/s41419-021-03830-5 (PMC8167120; doi:10.1038/s41419-021-03830-5)
Supplement: Supplementary file 2 — Supplementary Methods [file 41419_2021_3830_MOESM2_ESM.pdf]

Supplementary Methods

Image J Macro for quantification of DAPI-positive nuclei

```
dir1 = getDirectory("INPUT");
dir2 = getDirectory("OUTPUT");
setBatchMode(true);
list = getFileList(dir1);
for (i=0; i<list.length; i++)
   {
      open(dir1+list[i]);
      title = File.nameWithoutExtension ;
            run("Split Channels");
             saveAs("Tiff", dir2+title+"_DAPI.tif");
                                    close();
                                    close();

                              open(dir2+title+"_DAPI.tif");
                               run("Unsharp Mask...", "radius=500 mask=0.90");
                               run("Median...", "radius=10");
                               run("Make Binary");
                               run("Fill Holes");
                               run("Adjustable Watershed", "tolerance=9");
                               run("Analyze Particles...", "size=6-Infinity summarize");
                               saveAs("Tiff", dir2+title+"_DAPI_counts.tif");
                                    close();

}
setBatchMode(false);
selectWindow("Results");
saveAs("Results", output + filename + ".csv");
showMessage("You're awesome!");
exit();
```

Image J Macro for quantification of LC3-positive dots

```
dir1 = getDirectory("INPUT");
dir2 = getDirectory("OUTPUT");
setBatchMode(true);
list = getFileList(dir1);
for (i=0; i<list.length; i++)
   {
      open(dir1+list[i]);
      title = File.nameWithoutExtension ;
            run("Split Channels");
             saveAs("Tiff", dir2+title+"_LC3L.tif");
                                          close();
                                          close();



                                                                 run("Subtract Background...",
"rolling=60");
                                                                 setThreshold(75, 255);
                                                     run("Convert to Mask");
                                                     run("Watershed");
                                                     run("Analyze Particles...", "  summarize");
                                                               saveAs("Tiff", dir2+title+"_LC3_binary.tif");
                                                                 close();



}
setBatchMode(false);
selectWindow("Results");
saveAs("Results", output + filename + ".csv");
showMessage("You're awesome!");
exit();
```

Image J Macro for quantification of ATG16L1-positive dots

```
dir1 = getDirectory("INPUT");
dir2 = getDirectory("OUTPUT");
setBatchMode(true);
list = getFileList(dir1);
for (i=0; i<list.length; i++)
   {
      open(dir1+list[i]);
      title = File.nameWithoutExtension ;
            run("Split Channels");
             saveAs("Tiff", dir2+title+"_ATG16L.tif");
                                    close();
                                    close();

                                                      run("Gaussian Blur...", "sigma=3");
                                                      run("Subtract Background...", "rolling=8");

                                                      setThreshold(50, 255);
                                          run("Convert to Mask");
                                          run("Watershed");
                                          run("Analyze Particles...", "  summarize");
                                                saveAs("Tiff", dir2+title+"_ATG16L_binary.tif");
                                                      close();

}
setBatchMode(false);
selectWindow("Summary");
saveAs("Text", dir2+"Summary2.txt");
showMessage("You're awesome!");
exit();
```

Image J Macro for quantification of WIPI2-positive dots

```
dir1 = getDirectory("INPUT");
dir2 = getDirectory("OUTPUT");
setBatchMode(true);
list = getFileList(dir1);
for (i=0; i<list.length; i++)
   {
      open(dir1+list[i]);
      title = File.nameWithoutExtension ;
            run("Split Channels");
             saveAs("Tiff", dir2+title+"_WIPI2.tif");
                                        close();
                                        close();
                                        run("Gaussian Blur...", "sigma=3");
                                                              run("Subtract Background...", "rolling=6");
                                                              setThreshold(40, 255);
                                                run("Convert to Mask");
                                                run("Analyze Particles...", "  summarize");
                                                         saveAs("Tiff", dir2+title+"_WIPI2_binary.tif");
                                                              close();


}
setBatchMode(false);
selectWindow("Summary");
saveAs("Text", dir2+"Summary2.txt");
showMessage("You're awesome!");
exit();
```

Image J Macro for colocalization analysis of ATG16L1 & LC3 using Pearson's correlation coefficient after Costes automatic thresholding

```
dir1 = getDirectory("INPUT ");
dir2 = getDirectory("OUTPUT ");
setBatchMode(true);
list = getFileList(dir1);
for (i=0; i<list.length; i++)
    {
      open(dir1+list[i]);
      title = File.nameWithoutExtension ;

            //save channels
            getTitle();
            run("Split Channels");
            selectWindow("C3-"+title+".czi - "+title+".czi #1");
            close();
            selectWindow("C1-"+title+".czi - "+title+".czi #1");
            rename("C1");
            selectWindow("C2-"+title+".czi - "+title+".czi #1");
            rename("C2");

    run("JACoP ", "imga=[C1] imgb=[C2] pearson costesthr ica
costesrand=10-1-1000-0.001-0-false-true-true");
            close();
            close();
            close();
            close();
            close();
            close();
            close();
            close();

}
setBatchMode(false);
selectWindow("Log");
saveAs("Text", dir2+"Summary.txt");

            showMessage("JaCoP done!");
```
